# Allele distribution and phenotypic resistance to ciprofloxacin and gentamicin among extended-spectrum β-lactamase-producing *Escherichia coli* isolated from the urine, stool, animals, and environments of patients with presumptive urinary tract infection in Tanzania

**DOI:** 10.3389/frabi.2023.1164016

**Published:** 2023-06-05

**Authors:** Adam A. Mwakyoma, Benson R. Kidenya, Caroline A. Minja, Martha F. Mushi, Alison Sandeman, Wilber Sabiti, Mathew T. G. Holden, Stephen E. Mshana

**Affiliations:** ^1^ Department of Biochemistry and Molecular Biology, Catholic University of Health and Allied Sciences, Mwanza, Tanzania; ^2^ Department of Clinical Microbiology, Kilimanjaro Christian Medical Centre, Moshi, Tanzania; ^3^ Department of Microbiology and Immunology, Weill Bugando School of Medicine, Catholic University of Health and Allied Sciences, Mwanza, Tanzania; ^4^ School of Medicine, University of St Andrews, St Andrews, United Kingdom

**Keywords:** ESBL-producing *E. coli*, ESBL allele, non-beta lactam antibiotic, ciprofloxacin, gentamicin

## Abstract

**Background:**

Additional antimicrobial resistance to extended-spectrum β-lactamase (ESBL)-producing *E. coli* exhausts treatment options. We investigated allele distribution and resistance to ciprofloxacin and gentamicin among ESBL-producing *E. coli* isolates from the urine, stool, animals, and environments of presumptive urinary tract infection (UTI) patients, in order to gain a crucial insight toward devising prevention and control measures and treatment guidelines.

**Methods:**

Archived ESBL-producing *E. coli* isolates from the urine, stool, animals, and surrounding environments of presumptive UTI patients were retrieved. Antimicrobial susceptibility profiles for ciprofloxacin and gentamicin were done followed by multiplex Polymerase chain reaction (PCR) for *bla_CTX-M_*, *bla_TEM_*, and *bla_SHV_*, to determine ESBL allele distribution. Data were analyzed using STATA version 17.

**Results:**

A total of 472 confirmed ESBL-producing *E. coli* isolates from Mwanza 243 (51.5%), Kilimanjaro 143 (30.3%), and Mbeya 86 (18.2%) were analyzed. Of these, 75 (15.9%) were from urine, 199 (42.2%) from stool, 58 (12.3%) from rectal/cloaca swabs of animals, and 140 (29.7%) from surrounding environments. Out of the 472 ESBL-producing *E. coli*, 98.9% (467) had at least one ESBL allele. The most frequent allele was *bla_CTX-M_*, which was detected in 88.1% (416/472) of isolates, followed by the *bla_TEM_* allele, which was detected in 51.5% (243/472) of isolates. A total of 40.7% (192/472) of isolates harbored dual *bla_CTX-M_* + *bla_TEM_* alleles and only 0.2% (1/472) of isolates had dual *bla_CTX-M_* + *bla_SHV_* alleles, whereas 2.3% (11/472) of isolates had a combination of all three alleles (*bla_CTX-M_* + *bla_TEM_* + *bla_SHV_*). None of the isolates harbored a combination of *bla_TEM_* + *bla_SHV_* only. Resistance to ciprofloxacin and gentamicin was observed in 70.8% (334/472) and 46.0% (217/472) of isolates, respectively. There was a significant difference in the distribution of resistance to ciprofloxacin as well as gentamicin among ESBL-producing *E. coli* isolated from various sources (*p*-value < 0.001 and 0.002, respectively).

**Conclusion:**

Almost all ESBL-producing *E. coli* isolates carry *bla_CTX-M_*, *bla_TEM_*, and *bla_SHV_* either alone or in combination, with the most common allele being *bla_CTX-M._* The resistance to ciprofloxacin and gentamicin, which are frontline antibiotics for UTIs among ESBL-producing *E. coli*, is high. This implies the need to continually revise the local guidelines used for optimal empirical therapy for UTIs, and for continual research and surveillance using one health approach.

## Introduction

*Escherichia coli* is the main causative pathogen for urinary tract infections (UTIs) and has the greatest potential to acquire extended-spectrum β-lactamases (ESBLs) (Ahmed et al., 2015; Ayoyi et al., 2017; Karikari et al., 2021). The dissemination of ESBL-producing *E. coli* poses a significant public health threat, as the antibiotic resistance associated with it limits treatment options and challenges health systems (Jasser, 2006).

ESBLs comprise many plasmid-mediated derivatives such as *bla_TEM_*, *bla_OXA_*, *bla_SHV_*, and *bla_CTX-M_* (Nicolas-Chanoine et al., 2008; Peirano and Pitout, 2010). However, *bla_CTX-M_* has been the predominant ESBL allele worldwide (Cantón and Coque, 2006), including in Tanzania (Mshana et al., 2016; Seni et al., 2016). This group of ESBLs is associated with an extensive pattern of antimicrobial resistance to many antibiotics, including *β*-lactam agents such as penicillins, cephalosporins, monobactams, and carbapenems (Paterson and Bonomo, 2005; Rogers et al., 2011; Accogli et al., 2014; Cai et al., 2014; Johnson et al., 2015). In addition, over the last two decades ESBL-producing *E. coli* isolates have demonstrated an increased level of dual resistance to other frontline antibiotics such as aminoglycosides and fluoroquinolones (Meier et al., 2011; Mshana et al., 2011; Rogers et al., 2011). Several surveillance studies across Europe, North America, and South America have reported resistance to these antibiotics, ranging from 20% to 45% among uropathogenic *E. coli* isolates (Foxaman, 2010; Croxall et al., 2011). As most of these antibiotics are used to treat uncomplicated UTIs and complicated UTIs, which are the leading cause of increased UTI-related hospital visits, this increasing level of antimicrobial resistance of ESBL-producing *E. coli* to frontline antibiotics is of great concern. It threatens health systems by limiting the therapeutic choices used for treating UTIs and highlights the growing threat of the emergence of pan-drug resistance in ESBL-producing *E. coli* (Meier et al., 2011). At the time of writing treatment of UTIs is frequently initiated empirically (based on the standard treatment guideline), of which ciprofloxacin and gentamicin are recommended in (The United Republic of Tanzania Ministry of Health and Social Welfare, 2021). Having prior information regarding antimicrobial susceptibility profiles to frontline antimicrobial drugs for common causative pathogens, such as ESBL-producing *E. coli* in a particular setting, is essential to achieving the most effective empirical therapy as it will provide clinicians with the information required to facilitate the effective treatment and management of UTI patients (Dias Neto et al., 2003; Farajnia et al., 2009).

UTIs are among the most common bacterial infections acquired in community and hospital settings (Foxaman, 2010; Murray et al., 2022), and they are a main cause of hospital admissions that are associated with high morbidity, mortality, and economic costs (Gonzalez and Schaeffer, 1999; Foxman, 2002; Cove Smith and Almond, 2007; Murray et al., 2022). Pathogens causing UTIs can be acquired either endogenously or exogenously (Ayoyi et al., 2017), with about 87.0% of UTIs being endogenously acquired (Nielsen et al., 2014; Tandogdu and Wagenlehner, 2016). In addition, *E. coli* colonizing the gastrointestinal tract of humans and animals is described as being the main source of UTIs (Monstein et al., 2007; Jakobsen et al., 2012; Nielsen et al., 2014). Previous studies done in Tanzania reported the prevalence of ESBL-producing *E. coli* colonizing the gastrointestinal tracts of the adult population and animals in the community to be 16.5% and 20.8%, respectively (Mshana et al., 2016; Seni et al., 2016). Furthermore, in Tanzania, the evidence of ESBL-producing *E. coli* contaminating household latrines has been reported to be at 8.7% (Erb et al., 2018). It is also known that the distribution of ESBL alleles and their antibiotic susceptibility profiles, particularly to non β-lactam antibiotics, differ regionally (Mathai et al., 2001; Farrell et al., 2003). With this note, evaluation of common local ESBL allele distribution of *E. coli* strains circulating in the community among patients, their domesticated and farm animals, and surrounding environments is crucial in devising strategies to curb the spread of ESBL-producing *E. coli.* Therefore, this study investigated the allele distribution and antimicrobial resistance patterns of ciprofloxacin and gentamicin among ESBL-producing *E. coli* isolates from the urine and stool of presumptive UTI patients, their domesticated and farm animals, and their surrounding environments.

## Materials and methods

### Study design, period, and population

This was a laboratory-based cross-sectional study that utilized a total of 472 ESBL-producing *E. coli* isolates, which were selected from Gram-negative bacteria archived during the implementation of the Holistic Approach To Unravel Antibacterial resistance (HATUA) project. The HATUA project was conducted in three countries—Kenya, Tanzania, and Uganda—for the period of February 2019 to September 2020 (Asiimwe et al., 2021). In Tanzania, the HATUA project enrolled presumptive UTI patients selected from 10 health facilities in three regions (Mwanza, Mbeya, and Kilimanjaro). The health facilities included Kilimanjaro Christian Medical Center (KCMC), Kibosho District Designated Hospital, and Majengo Health Center for the Kilimanjaro region; Bugando Medical Center, Sekou-Toure Regional Hospital, Nyamagana District Hospital, Sengerema Designated District Hospital, and Makongoro Health Center for the Mwanza region; and Mbeya Regional Referral Hospital and Ifisi Designated District Hospital for the Mbeya region. Out of 472 isolates, 75 were from urine and 199 from stools of these presumptive patients, 58 were from rectal/cloaca swabs of their domesticated and farm animals (dogs, chickens, goats, cows, pigs, ducks, cats, and rabbits), and 140 were from the surrounding environments (bathrooms, toilets, and waste bins or dumping pits) of these presumptive UTI patients.

### Data collection

The information related to ESBL-producing *E. coli* isolates from study participants was retrieved from the pre-existing database of the HATUA project.

### Laboratory procedures and methods

#### Isolates recovery

The isolates were taken from cryovials, containing brain–heart infusion broth with 20% glycerol, stored in –80°C freezers. The isolates were then subcultured on sheep blood agar and incubated aerobically at 37°C for 18 to 24 h.

#### Antibiotic susceptibility testing

The resistance phenotypes for ESBL-producing *E. coli* from urine, stool, rectal swabs of animals, and environments were captured from the existing database of the HATUA project. Ciprofloxacin and gentamicin were tested using an agar dilution method for ESBL-producing *E. coli* originating from the environment and animals only. The agar dilution methods were determined according to established standard operating protocols based on methodology from the (Clinical and Laboratory Standards Institute, 2019). For gentamicin, the stock solution concentration of 40 mg/mL (40 µL) was incorporated into 1,000 mL of Mueller–Hinton media to make a final concentration of 8 µg/mL, while 10 mg/mL (50 µl) of stock solution of ciprofloxacin was incorporated into 1,000 mL of Mueller–Hinton media to make a final concentration of 0.5 µg/mL. The samples were inoculated onto media containing antibiotics and finally incubated at 37°C for 18 to 24 h.

#### DNA extraction

The boil lysate technique was used to extract bacterial DNA, as previously reported with a slight modification (Minja et al., 2021). Briefly, two colonies of overnight growth of bacteria from Mueller–Hinton agar were suspended into DNAse-free water, and, thereafter, mixed by vortexing and then boiled at 100°C in a water bath for 15 min. Tubes were centrifuged at 12,000 rpm for 10 min. The quality of DNA was determined using gel electrophoresis, whereas quantity was determined using Qubit^®^. Thereafter, 100 µL of the supernatant (DNA rich) was aliquoted into Eppendorf tubes for storage at –20°C for further PCR amplification and detection of ESBL alleles (*bla_CTX-M_*, *bla_TEM_*, and *bla_SHV_*).

#### Multiplex PCR amplification for detection of extended-spectrum β-lactamase alleles

A multiplex PCR was performed on a thermal cycler machine (T100™; Bio-Rad, Singapore) to amplify ESBL alleles (*bla_CTX-M_*, *bla_TEM_*, and *bla_SHV_*) using specific primers, as previous reported (Monstein et al., 2007) (**Table 1**). Briefly, 2 µL (≈ 30 ng) of DNA samples was added into PCR plates containing 12.5 µL of readily reconstituted master-mix (New England Biolabs) with 0.5 µL (500 µg) of each primer and then PCR water was added to make a final volume of 25 µL for the reaction mixture. Amplification conditions included an initial denaturation at 95°C for 15 min followed by 30 cycles of denaturation at 94°C for 30 s, annealing at 60°C for 40 s, and elongation at 72°C for 2 min. Then, a final elongation at 72°C for 10 min completed the process (Monstein et al., 2007).

**Table 1:** The details of PCR primer sequences and amplicon sizes.

Gene targets	Primer name	Primer sequences	Product size
*SHV*	SHV_F SHV_R	5′-ATGCGTTATATTCGCCTGTG-3′ 5′-TGCTTTGTTATTCGGGCCAA-3′	747 bp
*TEM*	TEM_F TEM_R	5 ′-TCGCCGCATACACTATTCTCAGAATGA-3′ 5 ′-ACGCTCACCGGCTCCAGATTTAT-3’	445 bp
*CTX-M*	CTX-M_U_F CTX-M_U_R	5′-ATGTGCAGYACCAGTAARGTKATGGC-3′ 5′-TGGGTRAARTARGTSACCAGAAYCAGCGG-3′	593 bp

#### Gel electrophoresis

The PCR products were visualized under UV illumination *via* gel electrophoresis using 1.5% ultrapure agarose gel (Thermo Fisher Scientific, UK) with a Tri-acetate-EDTA (TAE) buffer. Staining of the DNA fragments was carried out using Safe-Red dye (Safeview™Classic). The gels were run at 80 V for approximately 45 min. Standard DNA molecular weight markers were used: a 100-bp ladder. The ladder was visualized under UV light.

### Quality control

*Klebsiella pneumonia* ATCC 700603, *E. coli* ATCC 35218, and a clinical isolate of non-ESBL-producing *E. coli* were used as control strains. These control strains were used to check the performance of used media and antibiotic discs, as well as multiplex PCR experiments for the amplification and detection of ESBL alleles.

### Data management and statistical analysis

Data from isolates, such as identification number, isolate name, source of isolation, antimicrobial resistance pattern, and ESBL allele after PCR, were recorded in the logbook and then entered into the computer using Microsoft Excel^®^ 2018 (Microsoft Corporation, Redmond, WA, USA). Data were imported into STATA software version 17 (StataCorp, College Station, TX, USA) for analysis. Categorical variables were summarized using frequency and proportion (percent). To compare the difference in the proportion of distribution of resistance with ciprofloxacin and gentamicin we used a one-tailed two-sample proportion test. To determine the significance of the difference in the distribution of ciprofloxacin resistance as well as gentamicin resistance across various ESBL alleles and sources of *E. coli* isolation, we used Pearson’s chi-squared test or Fisher’s exact test where appropriate. In all analyses, the significance level was set at a *p*-value < 0.05.

### Ethics clearance

The study received ethics approval from the University of St Andrews, St Andrews, UK (No. MD14548, 10/09/19); the National Institute for Medical Research, Tanzania (No. 2831, updated 26/07/19); the Mbeya Medical Research and Ethics Committee (No. SZEC-2439/R.A/V.1/30); the Kilimanjaro Christian Medical College, Tanzania (No. 2293, updated 14/08/19); and the CUHAS/BMC Research Ethics and Review Committee (No. CREC/266/2018, updated on 02/2019).

## Results

### Isolates distribution

A total of 472 confirmed ESBL-producing *E. coli* isolates were retrieved for this study. These isolates were from Mwanza [243 (51.5%)], Kilimanjaro [143 (30.3%)], and Mbeya [86 (18.2%)]. Of these 472 isolates, 75 (15.9%) were from the urine of presumptive UTI patients, 199 (42.2%) were from the stool of these patients, 140 (29.7%) were from the surrounding environments, and 58 (12.3%) were from the domesticated and farm animals of these patients (**Table 2**).

**Table 2:** Distribution of 472 extended-spectrum β-lactamase (ESBL)-producing *Escherichia coli* isolates, by region and source.

Region	Urine	Stool	Environment	Animal	Total
	*n* (%)	*n* (%)	*n* (%)	*n* (%)	*N* (%)
Mwanza	27 (36.0)	116 (58.3)	80 (57.1)	19 (32.8)	**243 (51.5)**
Kilimanjaro	21 (28.0)	38 (19.1)	49 (35.0)	35 (60.3)	**14 (30.3)**
Mbeya	27 (36.0)	45 (22.6)	11 (7.9)	4 (6.9)	**86 (18.2)**
**Total**	**75 (15.9)**	**199 (42.2)**	**140 (29.7)**	**58 (12.3)**	**472 (100)**

GIT, gastrointestinal tract.

The bolded values indicates the row sum and column sum.

### Distribution of extended-spectrum β-lactamase alleles among extended-spectrum β-lactamase-producing *E. coli*

**Figure 1:**
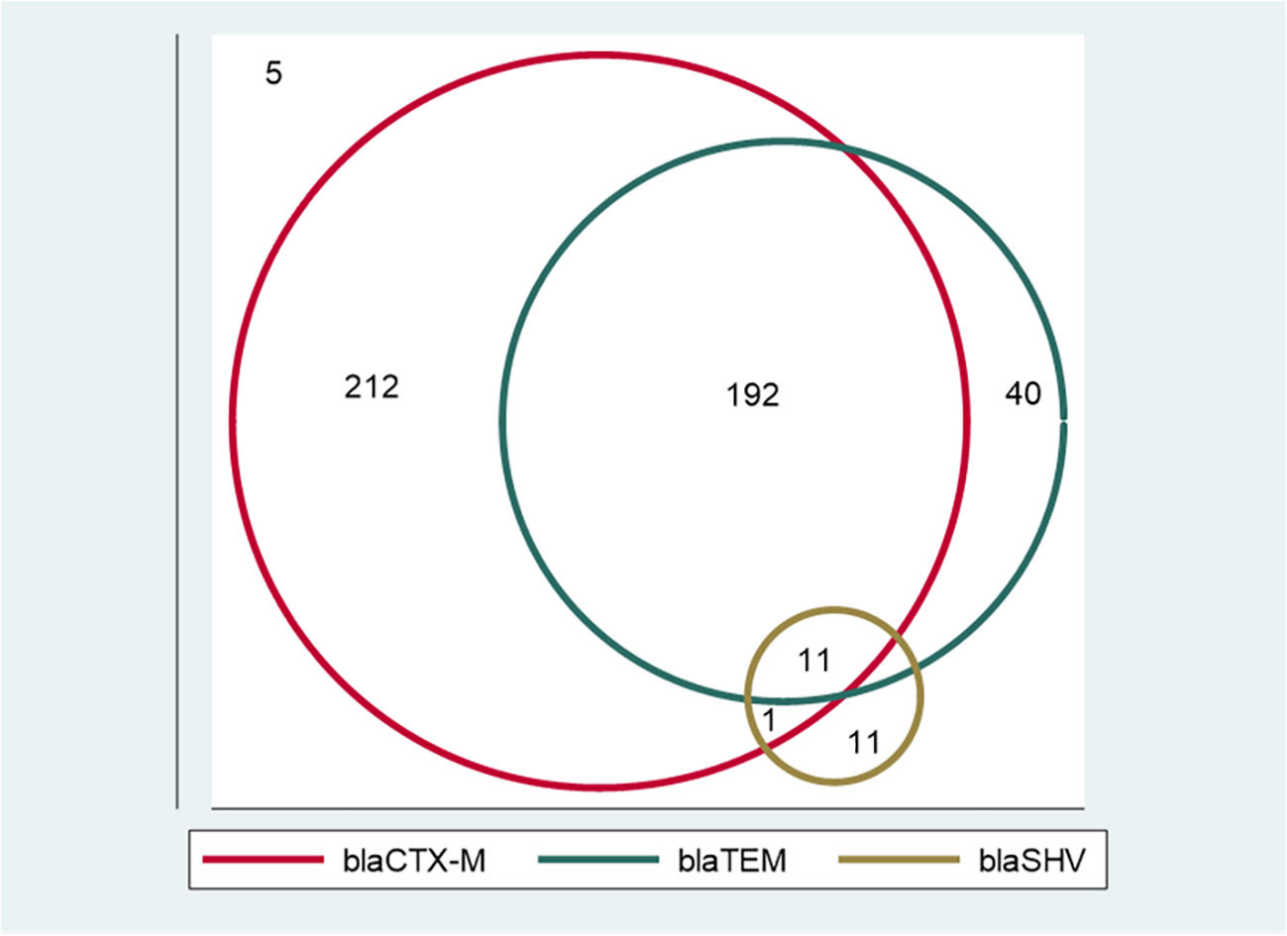
A Venn diagram showing the distribution of extended-spectrum β-lactamase (ESBL) alleles among ESBL-producing *E. coli.* Isolates with only one ESBL allele: 212 with *bla_CTX-M_*, 40 with *bla_TEM_*, and 11 with *bla_SHV_*. Isolates with dual ESBL alleles: 192 with *bla_CTX-M_* + *bla_TEM_*, and one with *bla_CTX-M_* + *bla_SHV_*. There were 11 isolates that had all three alleles (*bla_CTX-M_* + *bla_TEM_* + *bla_SHV_*). None of the isolates harbored a dual combination of *bla_TEM_* + *bla_SHV_* alleles. Five isolates did not harbor any of the tested ESBL alleles.

**Figure 2:**
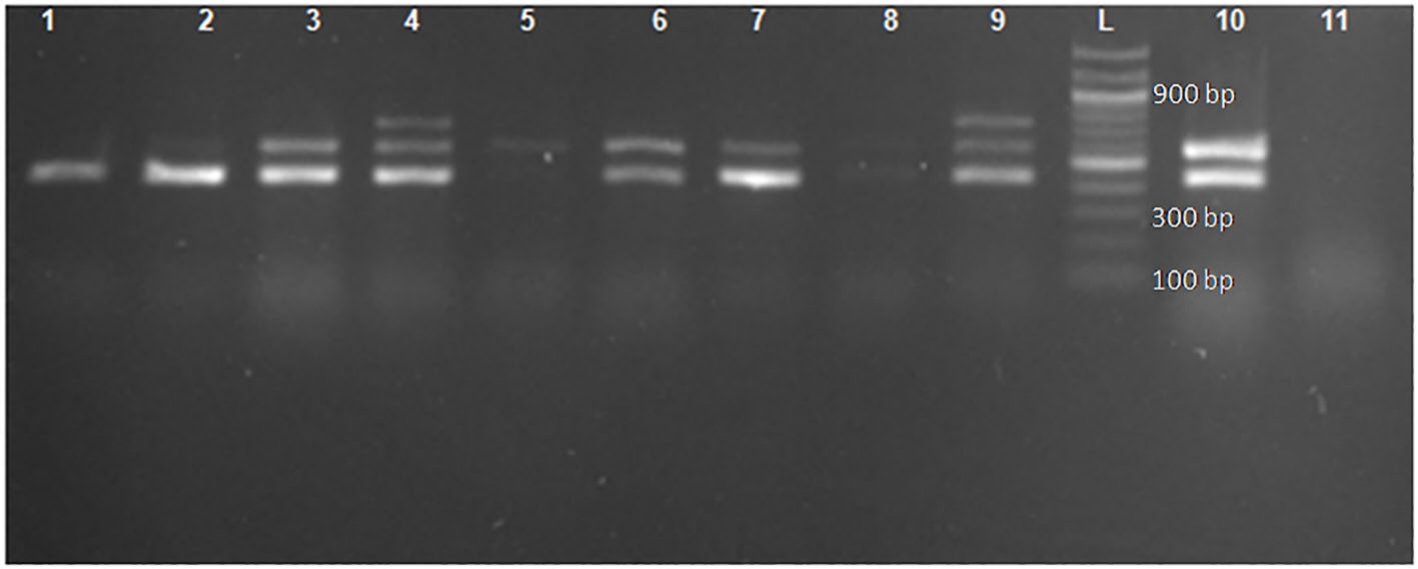
Gel image for the detection of genes encoding *bla_CTX-M_*, *bla_TEM_*, and *bla_SHV_* following multiplex PCR. Lane L: ladder 100 bp (New England Biolabs). Lane 2: the 445-bp PCR product of *bla_TEM_*. Lanes 3, 6, 7, and 10: the 445-bp and 593-bp PCR product of *bla_TEM_* and *bla_CTX-M_*, respectively. Lanes 4 and 9: the 445-bp, 593-bp, and 747-bp PCR products of *bla_TEM_*, *bla_CTX-M_*, and *bla_SHV_*, respectively. Lane 1: positive control *bla_TEM_* (*E. coli* ATCC 35218). Lane 11: negative control (*E. coli* clinical isolate non-ESBL).

Of the 472 phenotypically confirmed ESBL-producing *E. coli*, 98.9% (467) had at least one ESBL allele, and only five (1.1%) were negative for the tested ESBL alleles (*bla_CTX-M_*, *bla_TEM_*, and *bla_SHV_*). The most predominant allele was *bla_CTX-M_*, which was detected in 88.1% (416/472) of isolates, followed by *bla_TEM_* and *bla_SHV_* alleles, which were detected in 51.5% (243/472) and 4.9% (23/472) of isolates, respectively. A total of 55.7% (263/472) of isolates (212 with *bla_CTX-M_*, 40 with *bla_TEM_*, and 11 with *bla_SHV_*) harbored only one allele. A total of 40.7% (192/472) of isolates harbored *bla_CTX-M_* + *bla_TEM_* alleles, and only one (0.2%) isolate harbored *bla_CTX-M_* + *bla_SHV_* alleles, whereas 2.3% (11/472) of isolates harbored a combination of all three alleles investigated (*bla_CTX-M_* + *bla_TEM_* + *bla_SHV_*). Of note, none of the isolates harbored a dual combination of *bla_TEM_* + *bla_SHV_* (**Figure 1** and **Table 3**). All the positive ESBL alleles investigated showed a band on the gel electrophoresis following multiplex PCR amplification for the detection of ESBL alleles (*bla_CTX-M_*, *bla_TEM_*, and *bla_SHV_*) (**Figure 2**).

**Table 3:** Distribution of extended-spectrum β-lactamase (ESBL) alleles, by source of isolation, among 472 study *Escherichia coli* isolates.

ESBL allele	Sample source				Total, *N* (%)
	Urine, *n* (%)	Stool, *n* (%)	Environment, *n* (%)	Animal, *n* (%)	
*bla_CTX-M_*	42	82	48	40	**212 (44.9)**
*bla_TEM_*	5	13	16	6	**40 (8.5)**
*bla_SHV_*	0	10	1	0	**11 (2.3)**
*bla_CTX-M_* + *bla_TEM_*	27	86	69	10	**192 (40.7)**
*bla_CTX-M_* + *bla_SHV_*	0	0	1	0	**1 (0.2)**
*bla_CTX-M_* + b*la_TEM_* + *bla_SHV_*	1	6	2	2	**11 (2.3)**
Not detected	0	2	3	0	**5 (1.1)**
**Total**	**75**	**199**	**140**	**58**	**472 (100.0)**

The bolded values indicates the row sum and column sum.

### Phenotypic antimicrobial resistance to ciprofloxacin and gentamicin

Out of 472 ESBL-producing *E. coli*, resistance to ciprofloxacin and gentamicin was observed in 70.8% (334) and 46.0% (217) of isolates, respectively. The resistance to ciprofloxacin was significantly higher than that of gentamicin (*p*-value < 0.001; two-sample proportion test). Of note, 37.5% (177) of isolates were resistant to both antibiotics, whereas 20.8% (98) were sensitive to both drugs. Resistance to ciprofloxacin and gentamicin was highest for the isolates from urine, that is, 89.3% (67/75) and 56.0% (42/75), respectively. This was followed by the isolates originating from stool (fecal carriage), with 84.4% (168/199) for ciprofloxacin, and 55.0% (77/140) for isolates from the surrounding environments for gentamicin. There was a significant difference in the distribution of resistance to ciprofloxacin as well as to gentamicin across various sources of ESBL-producing *E. coli* (*p*-value < 0.001 and 0.002, respectively; Pearson’s chi-squared test). The lowest resistance to ciprofloxacin was observed in ESBL-producing *E. coli* originating from animals [31.0% (18/58)], whereas the lowest resistance to gentamicin was observed in isolates originating from stool samples [73/199 (36.7%)]. There was no significant difference in the distribution of ciprofloxacin and gentamicin resistance between various ESBL alleles (*p*-values 0.062 and 0.962, respectively; Fisher’s exact test) (**Tables 4**, **5**). Of note is that more than half [53.3% (40/75)] of isolates from the urine of presumptive UTI patients had dual resistance to ciprofloxacin and gentamicin, and this was significantly more than from other sources (*p*-value 0.003; Pearson’s chi-squared test) (**Table 6**).

**Table 4:** Distribution of ciprofloxacin resistance among various sources of isolation and extended-spectrum β-lactamase (ESBL) alleles.

Isolate characteristic	Ciprofloxacin		Total, *N* (%)	Pearson’s chi-squared (df)	*p*-value
	Resistant	Sensitive			
	*n* (%)	*n* (%)			
Source of isolation					
Animal	18 (31.0)	40 (69.0)	58	85.9656 (3)	< 0.001
Environment	81 (57.9)	59 (42.1)	140		
Stool	168 (84.4)	31 (15.6)	199		
Urine	67 (89.3)	8 (10.7)	75		
**Total**	332	135	472		
ESBL allele					
* bla_CTX-M_*	144 (67.9)	68 (32.1)	212	–*	0.062
* bla_TEM_*	26 (65.0)	14 (35.0)	40		
* bla_SHV_*	10 (90.9)	1 (9.1)	11		
* bla_CTX-M_ + bla_TEM_*	146 (76.0)	46 (24.0)	192		
* bla_CTX-M_ + bla_SHV_*	0 (0.0)	1 (100.0)	1		
* bla_CTX-M_ + bla_TEM_ + bla_SHV_*	6 (54.6)	5 (45.5)	11		
**Total**	332	135	472		

*p-value was calculated using Fisher’s Exact test.

df, degrees of freedom.

**Table 5:** Distribution of gentamicin resistance among various sources of isolation and extended-spectrum β-lactamase (ESBL) alleles.

Isolate characteristic	Gentamicin		Total, *N* (%)	Pearson’s chi-squared (df)	*p*-value
	Resistant	Sensitive			
	*n* (%)	*n* (%)			
Source of isolation					
Animal	25 (43.1)	33 (56.9)	58	14.7352 (3)	0.002
Environment	77 (55.0)	63 (45.0)	140		
Stool	73 (36.7)	126 (63.3)	199		
Urine	42 (56.0)	33 (44.0)	75		
**Total**	332	135	472		
ESBL allele					
* bla_CTX-M_*	96 (45.3)	116 (54.7)	212	–*	0.962
* bla_TEM_*	21 (52.5)	19 (47.5)	40		
* bla_SHV_*	5 (45.5)	6 (54.6)	11		
* bla_CTX-M_ + bla_TEM_*	88 (45.8)	104 (54.2)	192		
* bla_CTX-M_ + bla_SHV_*	0 (0.0)	1 (100.0)	1		
* bla_CTX-M_ + bla_TEM_ + bla_SHV_*	5 (45.5)	6 (54.6)	11		
**Total**	332	135	472		

*p-value was calculated using Fisher’s Exact test.

df, degrees of freedom.

**Table 6:** Distribution of extended-spectrum β-lactamase (ESBL) isolates with dual resistance to ciprofloxacin and gentamicin by source of isolation.

Isolate characteristic	Dual Resistance to ciprofloxacin and gentamicin		Total, *N* (%)	Pearson’s chi-squared (df)	*p*-value
	Yes	No			
	*n* (%)	*n* (%)			
Source of isolation					
Animal	15 (25.9)	43 (74.1)	58	13.9774 (3)	0.003
Environment	57 (40.7)	83 (59.3)	140		
Stool	65 (32.7)	134 (67.3)	199		
Urine	40 (53.3)	35 (46.7)	75		
**Total**	177	338	472		

df, degrees of freedom.

## Discussion

The findings from this study reveal that almost all studied ESBL-producing *E. coli* from the urine and stool (fecal carriage) of presumptive UTI patients, their domesticated and farm animals, and their surrounding environments possess *bla_CTX-M_*, *bla_TEM_*, and *bla_SHV_* either alone or in combination, with the most common allele being *bla_CTX-M_* and the most predominant dual combination of alleles being *bla_CTX-M_* + *bla_TEM_*. Furthermore, the resistance of ESBL-producing *E. coli* to frontline antibiotics, ciprofloxacin, and gentamicin, which are currently used to treat UTIs, was high. This emphasizes the need to continually revise the local guidelines used for optimal empirical therapy for UTIs, for proper control methods, and for further research to combat antibiotic resistance.

The findings from this study show that 98.9% of all ESBL-producing *E. coli* investigated had at least one ESBL allele tested (*bla_CTX-M_*, *bla_TEM_*, or *bla_SHV_*), either as one allele or in a combination of two or three of these ESBL alleles. Only 1.1% of the isolates were negative for the ESBL alleles investigated. These isolates could be more likely harboring other ESBL alleles, such as *bla_OXA_*, which was not tested in this study but has been reported elsewhere around the world including Tanzania. Nonetheless, it has been reported to have a very low prevalence in our settings (Marando et al., 2018; Abrar et al., 2019; Onduru et al., 2021). Our finding tallies with the findings from a similar study that reported that 3.4% of ESBL-producing isolates investigated were negative for these ESBL alleles (*bla_CTX-M_*, *bla_TEM_*, and *bla_SHV_*) (Mirkalantari et al., 2020). We investigated three ESBL alleles (*bla_CTX-M_*, *bla_TEM_*, and *bla_SHV_*), as they are the most common ESBL alleles circulating in community and hospital settings in Eastern, Central, and Southern African countries (Onduru et al., 2021). Our findings are in line with previous studies that showed that almost all of the ESBL-producing *E. coli* is driven by the *bla_CTX-M_*, *bla_TEM_*, and *bla_SHV_* alleles (Mshana et al., 2011; Mshana et al., 2016; Moremi et al., 2017; Marando et al., 2018; Mirkalantari et al., 2020; Kimera et al., 2021; Onduru et al., 2021). Our finding that *bla_CTX-M_* is the most common allele followed by *bla_TEM_* is supported by many previous and recent studies conducted among isolates from environments, animals, patients, and human carriers, which report the predominance of the *bla_CTX-M_* allele to range from 76.5% to 100% (Moremi et al., 2017; Marando et al., 2018; Abrar et al., 2019; Mirkalantari et al., 2020; Kimera et al., 2021; Onduru et al., 2021). This predominance can be explained by the fact that the conjugative plasmid-carrying *bla_CTX-M_* allele is highly effective at being transferred and has been reported as the most frequently and successfully transferred allele through horizontal gene transfer (Cantón and Coque, 2006; Mshana et al., 2009; Minja et al., 2021). The spread of *bla_CTX-M_* allele is causing rapid, important, and unpredictable changes in the epidemiology of antibiotic resistance.

The most common combination of two alleles was *bla_CTX-M_* + *bla_TEM_* at 40.7%; the next most common combination was *bla_CTX-M_* + *bla_SHV_*, with a distant 0.2%. Similarly, the occurrence of dual ESBL alleles in the genes was common elsewhere, with the combination of *bla_CTX-M_* + *bla_TEM_* alleles being the most common (Sah et al., 2021; Silago et al., 2021). These genes are often present in large plasmids and are capable of conferring resistance to the organisms (Lee et al., 2012). Acquisition and transferability of ESBL genes are of particular importance, as these ESBL-encoding genes are often located in promiscuous plasmids (van Duijkeren et al., 2018). This property of ESBL genes enables their exchange between bacteria, and favors transmission between animals and humans (Brolund and Sandegren, 2016). For this reason, the distribution for all combinations of ESBL alleles was expected. Surprisingly, in our study there was no ESBL-producing *E. coli* with a combination of *bla_TEM_* + *bla_SHV_*. Furthermore, we observed only 2.3% of the ESBL-producing *E. coli* isolates with all three alleles (*bla_CTX-M_*, *bla_TEM_*, and *bla_SHV_*). These findings raise the thought of a genetic preponderance of order and dynamics in the combination, transmission, and acquisition of these genes for ESBL production. Further genome-wide studies are warranted to unravel the plausibility of this hypothesis.

Ciprofloxacin and gentamicin are frontline antimicrobials used to treat UTIs. Extra resistance to frontline non-β-lactam antibiotics, such as ciprofloxacin and gentamicin, to ESBL-producing *E. coli*, limits the therapeutic options to treat UTIs. Our findings that 70.8% and 46.0% of ESBL-producing *E. coli* are resistant to ciprofloxacin and gentamicin, respectively, are alarming. This result is similar to studies done among animals, street children, and patients in Dar es Salaam and Mwanza in Tanzania (Seni et al., 2016; Manyahi et al., 2017; Moremi et al., 2017; Kimera et al., 2021). This high prevalence is explained by the fact that these antibiotics are used as frontliners to treat uncomplicated and complicated UTIs, complicated UTIs being the leading causes of UTI-related increases in hospital visits. This increasing level of antimicrobial resistance of ESBL-producing *E. coli* to frontline antibiotics is of great concern as it highlights the growing threat of the emergence of pan-drug resistance in ESBL-producing *E. coli* (Meier et al., 2011).

Furthermore, the prevalence of ciprofloxacin and gentamicin resistance among ESBL *E. coli* is higher than the reported prevalence of ciprofloxacin and gentamicin reported in non-ESBL *E. coli* in our setting, which ranges from 48.6% to 62.7% and from 14.4% to 17% for ciprofloxacin and gentamicin, respectively (Msanga et al., 2022; Mtemisika et al., 2022). This relatively high resistance rate to fluroquinolones and aminoglycosides to ESBL-producing bacteria compared with non-ESBL-producing bacteria might be due to the co-existence of the ESBL genes with those conferring resistance to fluroquinolones and aminoglycosides in the same large plasmid (Lee et al., 2012). In this study, more than one-third of the isolates were resistant to both ciprofloxacin and gentamicin; this is worrisome as it limits treatment options in the case of acute pyelonephritis and urosepsis, as per standard treatment guidelines in the study setting. This could be due to the presence of the plasmid-mediated genes such as *aac (6′)-Ib-cr*, which encodes aminoglycoside acetyltransferase that induces resistance against aminoglycosides and fluoroquinolones simultaneously (Rasoulinasab et al., 2021).

The observed levels of high resistance to ciprofloxacin compared with gentamicin could be attributed to the fact that ciprofloxacin is an orally administered antibiotic and is among the more easily obtained over-the-counter antibiotics, whereas gentamicin is administered *via* injection, which therefore prevents it from being misused by non-health personnel. The treatment of UTIs is frequently initiated empirically; if a patient has ESBL-producing *E. coli*, ciprofloxacin and gentamicin will be more likely to end up with treatment failure. Further studies to assess other non-beta lactam antibiotics to treat ESBL-producing *E. coli* are warranted in achieving the most effective empirical therapy, as they will provide clinicians with the information required to facilitate the effective treatment and management of UTI patients (Dias Neto et al., 2003; Farajnia et al., 2009).

We found significant differences in the distribution of ciprofloxacin resistance across various sources of isolation of the ESBL-producing *E. coli*. The highest resistance to ciprofloxacin was observed from ESBL-producing *E. coli* isolated from the urine and stool of presumptive UTI patients, as well as from isolates from the environment as compared with the isolates from the animals. Our finding tallies with other studies in Africa that reported ciprofloxacin resistance among ESBL-producing isolates from humans to range from 46.3% to 85.5% (Moyo et al., 2010; Meier et al., 2011; Mirkalantari et al., 2020). This finding could be attributed to the fact that ciprofloxacin is more commonly used as the frontline drug to treat UTIs in humans than in animals. In addition, these presumptive UTI patients could have a reckless habit of contaminating their environment *via* their urine and stools. On the other hand, we found a significant difference in the distribution of gentamicin resistance across various sources of isolates of ESBL-producing *E. coli*. The highest level of resistance to gentamicin was observed from ESBL-producing *E. coli* isolated from the urine of presumptive UTI patients followed by those isolates from the environment, as compared with the isolates from the stool of patients with presumptive UTI (fecal carriage). Studies that compare the significance of the difference in the distribution of gentamicin as well as ciprofloxacin resistance among ESBL-producing *E. coli* in Africa are limited. However, our prevalence of gentamicin resistance for ESBL-producing *E. coli* from urine is similar to a study done in Dar Es Salaam, Tanzania (Manyahi et al., 2017). This finding could be attributed to the fact that gentamicin is mainly used for the treatment of UTIs and urosepsis and its use for domesticated and farm animals is less than that for humans; hence, resistance to isolates from stool is minimal.

In conclusion, almost all ESBL-producing *E. coli* isolates from urine and stool of presumptive patients of UTI, their animals, and their environment harbor *bla_CTX-M_*, *bla_TEM_*, and *bla_SHV_* either alone or in combination, with the most common allele being *bla_CTX-M_*. The most common allele combination was *bla_CTX-M_* + *bla_TEM_*. Higher resistance of ESBL-producing *E. coli* to current frontline antibiotics (ciprofloxacin and gentamicin) to treat UTIs than in non-ESBL-producing isolates emphasizes the need to continually revise the local guidelines used for optimal empirical therapy for UTIs and it calls for coordinated efforts to address the growing ESBL predicament. Further genome-wide studies are warranted to unravel the genetic dynamics and interplay in the transmission and acquisition of ESBL genes.

## Data availability statement

The raw data supporting the conclusions of this article will be made available by the authors, without undue reservation.

## Ethics statement

The studies involving human participants were reviewed and approved by the Joint Catholic University of Health and Allied Sciences – Bugando Medical Center Research and Ethics Committee. The patients/participants provided their written informed consent to participate in this study. The animal study was reviewed and approved by the Joint Catholic University of Health and Allied Sciences – Bugando Medical Center Research and Ethics Committee. Written informed consent was obtained from the owners for the participation of their animals in this study.

## Author contributions

AM, CM, BK, and SM designed the study. AM, CM, and MM carried out the experiments. BK, CM, and MM analyzed the results. AM, CM, and BK wrote the manuscript. WS, AS, MH, MM, SM, and BK critically reviewed the manuscript. All authors contributed to the article and approved the submitted version.
